# Cardiovascular Nutrition Beyond BMI: Integrating Malnutrition Assessment with Inflammation

**DOI:** 10.3390/nu17243955

**Published:** 2025-12-18

**Authors:** Joanna Popiolek-Kalisz, Grzegorz Kalisz

**Affiliations:** 1 Department of Clinical Dietetics, Medical University of Lublin, ul. Chodzki 7, 20-063 Lublin, Poland; 2 Department of Cardiology, Cardinal Wyszynski Hospital, al. Krasnicka 100, 20-718 Lublin, Poland; 3 Department of Bioanalytics, Medical University of Lublin, ul. Jaczewskiego 8b, 20-090 Lublin, Poland; grzegorz.kalisz@umlub.edu.pl

The constant advances being made in cardiovascular (CVD) medicine highlight its holistic nature, drifting away from strict traditional approaches. The Special Issue “Nutritional Aspects of Cardiovascular Disease Risk Factors” resulted in ten studies at the frontiers of cardiology and dietetics focusing on the application of function-centered understanding of nutritional status. The contributions collectively support a clinical paradigm in which malnutrition is identified early, monitored longitudinally, and treated as a core determinant of prognosis, while an upstream, modifiable inflammatory substrate, shaped by diet quality and health beliefs, is addressed in parallel. The full spectrum of those relationships ranges from dietary patterns that influence disease development, through the cognitive frameworks that shape health behaviors, to the critical assessment of nutritional status in established cardiovascular disease and its prognostic implications.

Malnutrition, beyond body mass index (BMI), in CVD patients is prevalent, underrecognized, and prognostically powerful, yet BMI neither captures the qualitative deficits in intake nor reflects cellular integrity. It refers to the pivotal role of diet quality and inflammation in the work by Mrozik et al. that addresses simplification of beliefs on plant-based diets [1]. This nuanced perspective emphasizes that the protective effects of plant-based nutrition depend not only on what is excluded but on what is included and the overall dietary pattern quality. This observation confirms large prospective analyses that demonstrated that ultra-processed food intake is associated with increased CVD risk [2]. Moreover, if a plant-based model is implemented, the direction and magnitude of cardiovascular protection depend on whether the plant-based pattern is rich in minimally processed, nutrient-dense foods versus refined grains, sugars, and other lower-quality plant-derived products [3]. This is particularly important, as pro-inflammatory dietary potential has been associated with CVD outcomes in large U.S. cohorts [4] and a meta-analysis [5]. This is further elaborated by Lopez de Coca et al., who systematically examined the relationship between the Dietary Inflammatory Index and various dietary patterns [6]. It concludes that adherence to anti-inflammatories potentially modulates therapeutic needs, not only by reduction in CVD risk. That is why plant-based models are recommended in cardiovascular prevention [7,8,9].

While diet quality influences disease development, the issue of malnutrition in CVD patients is an underrecognized and clinically significant phenomenon. Świątoniowska-Lonc et al. document the prevalence of malnutrition among heart failure patients and demonstrate its substantial impact on treatment effectiveness, complication rates, and hospitalization duration [10]. Their center’s experience reinforces that nutritional status should be considered a vital sign in cardiovascular care, not an ancillary concern.

Similar observation was presented by Czaja-Stolc in dialysis patients, examining the complex interplay between nutritional status, uremic toxins, and metabo-inflammatory biomarkers as predictors of two-year CVD mortality [11]. This particular population presented high CVD mortality, and the integration of nutritional parameters, biomarkers of inflammation, and uremic toxin levels offers a more comprehensive risk stratification approach.

The prognostic significance of nutritional status needs reliable, practical assessment methods. This was the topic of two reviews in this Special Issue. Jarosz et al. provided a systematic overview of nutritional status assessment tools specifically for CVD patients [12]. Malnutrition, despite its prevalence and prognostic value, remains underrecognized, particularly in patients with fluid overload and sarcopenic obesity. It shows the need for multidimensional assessment approaches in cardiovascular populations. Similar observations were presented in a meta-analysis by Fărcaș et al. on the Controlling Nutritional Status (CONUT) score [13]. It was concluded to be a readily available, cost-effective tool providing valuable prognostic information on predicting mortality in acute and chronic heart failure. The integration of basic biochemical parameters into composite scores was again proven to enhance risk stratification without requiring specialized equipment or extensive resources. Similar observations were made regarding the Geriatric Nutritional Risk Index and phase angle (PhA) as predictors of heart failure mortality as well [14,15]. Cardiac surgery patients can benefit not only from a cardiovascular risk assessment but also from advanced approaches employing methods beyond BMI analysis such as PhA and bioelectrical impedance analysis (BIA), reflecting cell condition [16,17]. In a study by Popiolek-Kalisz et al., it was demonstrated that PhA serves as a reliable, non-invasive marker of nutritional status with prognostic value in surgical cardiovascular populations [18]. The practical applicability of BIA at the bedside makes it particularly attractive for routine clinical use. International consensus has also moved toward standardized operationalization of malnutrition, most prominently via the Global Leadership Initiative on Malnutrition (GLIM) framework [19], which formalizes phenotypic and etiologic criteria and underscores that malnutrition can be present across the BMI spectrum. This aligns with contemporary prevention guidelines that position dietary patterns as foundational therapy in cardiovascular risk management [20], thereby linking nutritional phenotyping to actionable lifestyle targets rather than to weight alone.

The path forward is integrative rather than competitive in research ideas. Malnutrition and inflammation are distinct axes that converge on risk and recovery. Measuring the first while modifying the second is both mechanistically coherent and operationally feasible. By embedding functional nutritional phenotyping into routine cardiology, cardiac surgery, and nephrology, and by elevating diet quality and belief-informed counseling to topmost priorities [21,22], cardiovascular teams can bridge mechanisms and outcomes and improve the lives of patients now, while laying the foundation for phenotype-guided trials that will define the next chapter of cardiovascular nutrition. Implementation science must be engaged to determine how nutritional screening, assessment, and intervention can be routinely incorporated into CVD care workflows, ensuring that validated tools and evidence-based strategies actually reach patients in clinical practice, and using recognized frameworks [23,24]. Finally, human translational studies utilizing advanced omics technologies, metabolic tracers, and other sophisticated methodologies are needed to bridge the gap between preclinical mechanistic insights and clinical outcomes, thereby identifying novel therapeutic targets and biomarkers for personalized nutritional interventions.
